# Evidence in practice: implementing KAT in indigenous health services

**DOI:** 10.3389/fpsyt.2026.1722573

**Published:** 2026-04-22

**Authors:** Jean Jacque Lovely, Hiedi Yardley, Reverdi Darda, Quintina Bearchief-Adolpho, Lisa Kemp, Charlene Brough, Vanessa Doore, Jennifer Kohlhammer, Andrew Charrette

**Affiliations:** 1 AtmaCena Psychedelic Healthcare Solutions, Edmonton, AB, Canada; 2 Siksika Health Services, Siksika, AB, Canada

**Keywords:** culturally integrated care, evidence in practice, indigenous mental health, ketamine assisted therapy, psychedelic assisted therapy

## Abstract

Ketamine-Assisted Therapy (KAT) presents a promising alternative for addressing mental health challenges, particularly in treatment-resistant conditions, yet little exists in the literature guiding its implementation in an Indigenous context, for Indigenous participants, or describing culturally adapted delivery models. This paper presents insights and lessons learned from a collaborative pilot program between Siksika Health Services and ATMA CENA to design and deliver a culturally responsive KAT program within the Siksika First Nation in Alberta Canada. The initiative aimed to explore the feasibility and therapeutic impact of KAT in an Indigenous healthcare setting, while also being conscious of cultural relevance and opportunities for continued clinical and quality improvement of the program. The pilot followed a five-phase approach: collaboration, knowledge acquisition, lived experience, data collection, and follow-up. Recruitment resulted in 6 participants completing care (3 Indigenous and 3 non-Indigenous). Findings demonstrated notable improvements in symptoms of depression, anxiety, and PTSD, with participants reporting increased emotional regulation and stronger cultural connections. Cultural elements including shared meals, traditional decor and blankets, community orientation, and a mid-program break for cultural events, were central to participant reported safety, trust, and meaning making. Notably, the Indigenous and non-Indigenous participant groups, who were treated together, reported comparable gains in safety, trust, and mental, emotional, and spiritual well-being. These shared outcomes suggest the model may hold relevance for reducing inequities in group KAT delivery. Challenges and lessons learned included need to address stigma and systemic influences experienced by Indigenous participants, barriers affecting timely intention setting and integration therapy, and overcoming logistical barriers when working in rural First Nation environments. This pilot program implementation underscores the importance of culturally responsive mental health interventions and highlights key considerations for expanding psychedelic-assisted therapies in Indigenous communities.

## Introduction: the challenge and the opportunity

The disparity in health outcomes for Indigenous populations in Canada is well-documented (1, 2). The lasting effects of colonialism, residential schools, and the ongoing impact of intergenerational trauma and systemic oppression have contributed to significant mental health challenges within these communities (2). Indigenous people, both on and off reserve, experience higher rates of suicide, particularly among youth, as well as elevated incidences of anxiety, depression, post-traumatic stress disorder (PTSD), and substance use disorders compared to the non-Indigenous population (3, 4). Conventional mental health interventions have often failed to adequately address the complex needs of Indigenous communities. Ketamine-Assisted Therapy (KAT) has emerged as a promising alternative, particularly for treatment-resistant conditions (5, 6). However, access to this care is limited in Canada and little has been shared informing its provision for Indigenous people (7).

The Siksika First Nation in Alberta faces a high prevalence of the noted mental health challenges. Despite the comprehensive and holistic services provided by Siksika Health Services (SHS), including mental health care aimed at addressing complex trauma, there is a recognized need for alternative approaches. Traditional and conventional treatments have shown limited effectiveness for some individuals, prompting the exploration of innovative therapies that could better meet their needs.

Psychedelic-assisted therapy is gaining traction globally as a promising treatment for mental health conditions that are resistant to conventional therapies, such as anxiety, depression, PTSD, and substance use disorders (8, 9). In Canada, the legal framework for accessing psychedelic medicines has been expanding, with Health Canada’s Special Access Program (SAP) providing avenues for the use of psilocybin and MDMA in mental health treatments for participants with serious conditions in instances where other therapies have failed (10–12). Unique in the country, Alberta has taken a leading role by becoming the first province to regulate and set service standards for psychedelic drug treatments including ketamine in addition to those accessible by the SAP under the Mental Health Services Protection Act (13). While some recent efforts are underway to explore psychedelic assisted therapy as a tool to address mental health needs in the Indigenous population, little is available regarding how to design, develop, and provide a program of psychedelic therapy for this population.

Ketamine-assisted therapy is an effective, safe, and clinically proven for mental health conditions (14–16). The potential of ketamine-assisted therapy (KAT) to serve as an effective treatment for the mental health challenges faced by the Siksika community members presents a unique opportunity. By integrating KAT within the culturally sensitive context of Siksika Health Services, there is a possibility to offer a therapeutic option that is not only innovative but also accessible, safe, sustainable, and aligned with the traditional values and healing practices of Siksika Nation.

In response to the pressing need for effective, culturally integrated mental health interventions, Siksika Health Services (SHS) partnered with ATMA CENA to pilot a KAT program within the Siksika First Nation. Both organizations share a common mission to address the mental health crisis and the disproportionate impact on the Indigenous population. Therefore, the pilot aimed to explore the feasibility of KAT within Indigenous healthcare frameworks, evaluate its therapeutic impact, and explore how traditional healing practices could be integrated into its administration.

## Context: a purposeful partnership

Siksika Nation is situated in Sothern Alberta and the surrounding territory which spans the province of Saskatchewan and into the state of Montana. It is part of the Siksikaitsitapi – Blackfoot Confederacy which includes Kainai Nation, Piikani Nation, and Aamskapi Piikani. The population of Siksika Nation is approximately 7800 +. The mental health and addiction challenges facing the nation must be understood within the broader historical, social, and political context. The legacy of colonialism, including the trauma inflicted by residential schools and other forms of systemic oppression, has left deep scars on Indigenous communities across Canada (17). These historical injustices have contributed to the high rates of mental health challenges, substance use, and suicide observed among Indigenous populations today (2).

Siksika Health Services was established in 1970 to offer comprehensive health services that integrate traditional Indigenous healing with modern medical practices. The organization provides a wide range of quality health and well-being services including mental health services aimed to help individuals to address their complex trauma, but significant challenges remain in addressing the deep-rooted psychological trauma affecting the community.

ATMA CENA is a mental health services organization purpose-built to increase mental well-being through accessible, high-quality alternatives to traditional treatments. ATMA CENA’s expertise in psychedelic assisted therapy provided the clinical foundation for the development of a pilot project ensuring treatment protocols adhered to evidence, regulation in the province, and the highest standards of quality and safety.

Recognizing the need for innovative mental health interventions, SHS partnered with ATMA CENA to explore KAT’s potential. This initiative was designed with respect for the Siksika Way of Life, integrating Indigenous values such as community relationships, respect for traditional knowledge, and alignment with spiritual practices. The pilot was developed in close collaboration with local healthcare providers, ensuring that it addressed community needs and was culturally relevant.

## Treatment program: project design, clinical design and treatment delivery method

The aim of this project was to design and implement a pilot program that could provide a new therapeutic option for the Siksika community. Initial relationship building and project planning meetings occurred over a year to develop a treatment program that was well-suited for the Siksika community and aligned with the concept of lived experience program design. Lived experience program design derives benefit by embedding and aligning three key principles in the process:

• Inclusion in the process is a right and socially just, “nothing about us without us (18)”;

• Ensuring inclusion improves design, analysis and meaning making as the interpretation includes the perspective from within;

• Through inclusive participation we all grow in awareness, knowledge, and capability (19, 20).

This approach, referred to by the partnering teams as “moving at the speed of trust,” allowed for transparency, engagement, collaboration and the co-creation of a culturally responsive model. By integrating Indigenous perspectives into program design, the initiative prioritized the needs of the SHS team, SHS staff, and collaborators, ensuring that participants felt comfortable and supported from the outset and that the pilot design was driven by them and for them. As Such the SHS team selected a KAT pilot model that provided treatment to SHS-identified staff who met clinical criteria. This was deemed a “heal the healers” approach with the long-term goal of expanding access to the broader Siksika Nation community. The program included both Indigenous and non-Indigenous staff members, participating together in a shared therapeutic setting. The pilot’s objectives were to: offer an alternative mental health service for individuals experiencing trauma, depression, anxiety, or substance use concerns; provide KAT to six clinically appropriate participants over a four to six week period; integrate culturally relevant practices into the therapeutic process and overall KAT framework; increase capacity and awareness among local healthcare providers regarding the safe and effective delivery of KAT; and evaluate participant outcomes and experiences to inform future service development and implementation within Siksika Nation.

5 Phased-Project Approach

The project followed five key phases: collaboration, knowledge acquisition, lived experience and understanding, data collection and evaluation, and follow-up and future planning.

Phase 1 collaboration: focused on co-creation and program development, ensuring alignment with the Siksika Way of Life and addressing ethical, safety, and regulatory considerations. Through a series of collaborative working sessions, the SHS and ATMA CENA teams worked together to share ideas, discuss strategies, explore evidence and treatment options, and determine creative solutions.

Phase 2 knowledge acquisition: involved knowledge acquisition and awareness building through educational sessions and provider training workshops detailing the concept of psychedelic assisted therapy, the status of care in Canada and in particular Alberta, the evidence supporting ketamine assisted therapy, and approaches for delivery of the care. The ATMA CENA team hosted 3, 1–2-hour sessions, remaining present until all participants had their questions and concerns addressed. Session participants (15–25 participants per session) were invited by the SHS Project Team recognizing that some were clinicians who traveled into the community to work, however many resided in the community and represented the breadth of community stakeholders including community elders. In addition, following this education sessions, the SHS team sought wisdom by connecting with trusted Elders and well-respected nation members in the community to inform moving forward into phase three.

Phase 3 lived experience and understanding: drew from the SHS teams experience and emphasized practical implementation, whereby the SHS team chose to have a small cohort of SHS staff participate in the KAT treatment sessions and to design the group treatment adhering to clinical evidence and Indigenous healing traditions. The decision to have a “heal the healers” approach was initiated by the staff themselves, when they recognized in the education sessions that they fit the criteria for KAT. Their request was then supported by the SHS leadership, with the intent that their experience would best inform planning and decision making for the pilot and future programming for SHS.

Phase 4 data collection and evaluation: consisted of data capture, review, and evaluation to measure outcomes and capture feedback. This was conducted utilizing psychometric assessments (Patient Health Questionnaire – PHQ-9, Generalized Anxiety Disorder Scale – GAD-7, and PTSD Checklist for Civilians – PCL-5). Psychometrics were completed prior to treatment and in post treatment follow-up; participant surveys completed at the end of the final integration session; and feedback opportunities with the project team as part of the project delivery. Additionally, the clinical team who provided care was given opportunity to share their observations and the feedback shared by participants during the delivery of medicine sessions and in the integration sessions.

Phase 5 follow-up and future planning: involved sessions conducted with the SHS team and ensured continued support for pilot participants through continued integration therapy and peer networks. Together the teams identified next steps and explored options and opportunities for continued partnership and future programming.

### Inclusion of critical components for KAT

The KAT treatment delivery method was built recognizing research that details critical factors which influence the success of psychedelic assisted therapeutic treatments (PAT) (11, 21, 22). These include preparation - set, the therapeutic environment - setting, the role of the therapist – therapeutic alliance, and the importance of integration.

**Set:** The planning and preparation stage, often referred to as “set” or mind-set, plays a foundational role in ensuring a positive therapeutic experience (23). As such, individual one-on-one intention setting therapy sessions were held by a trauma-informed psychologist with experience working within Indigenous first nation communities. The therapist met with each participant in advance of the group medicine sessions and attended the group orientation prior to commencing the treatment process This was done to build rapport, trust, and empathy with participants individually and as a group to assist with setting therapeutic intentions and to prepare participants for the experience.

Setting: The therapeutic setting, sometimes referred to as the “container” in psychedelic therapy, is another crucial element influencing treatment outcomes (15). The Siksika Health Services team carefully curated their own treatment environment for medicine sessions to reflect the values and aesthetics of the Siksika Way of Life. They chose the location and the treatment room. The location was off-site from the SHS main operations building, located in a smaller, recently renovated, comfortable support location on the nation. The treatment room was set-up and designed to be as calming and comfortable as possible, incorporating natural elements such as plants, artwork, and relaxing color schemes to create a sense of peace and familiarity. Reclining chairs, warm traditional blankets, weighted blankets, and soft lighting further enhanced the experience. Participants were also encouraged to bring their own comfort items on medicine days to provide additional comfort and safety during the treatment session. Examples of comfort items included: pillow, blanket, small personal item of remembrance to family or of personal meaning, and medicine bundle.

Additionally, participants were introduced to the nursing team and therapist during an orientation session that included a shared meal, which fostered warmth, trust, and transparency. A sharing circle format was introduced during the orientation to check-in individually with the participants. This allowed each participant, leadership team, and the clinical team to openly express their own feelings/reflections about being together for the KAT pilot. By establishing a safe and welcoming space from the outset and through-out, the project addressed the importance of environmental factors in psychedelic-assisted therapy.

Therapeutic alliance: The relationship between the therapist and the participant, known as the therapeutic alliance, is one of the most significant predictors of positive treatment outcomes (24). Therapeutic factors such as empathy, congruence, and positive regard are fundamental to the success of KAT (25). The therapists and clinical team in this project were carefully selected and trained to create a therapeutic alliance based on trust, respect, and cultural sensitivity. Opportunities to meet and get to know the therapists and care team were incorporated into the program design.

Integration: Psychedelic integration therapy involves working with a trained therapist to unpack and make meaning of a prior psychedelic experience and bring the wisdom into the present (26). Research holds that structured integration therapy sessions, held within ideally 48 and up to 72 hours after treatment, are essential for maximizing long-term benefits (27, 28). The integration phase was prioritized in this pilot, ensuring that each participant had scheduled follow-up therapy sessions after each treatment and that they were given the option to have these sessions on their nation. These sessions provided a flexible and structured space for participants to process their experiences and incorporate their insights into daily life. The inclusion of a therapist familiar with traditional healing methods as well as modern psychotherapeutic practices, allowed for a holistic integration approach inclusive of Indigenous perspectives and practices.

### Clinical design and treatment delivery method

**Figure 1:**
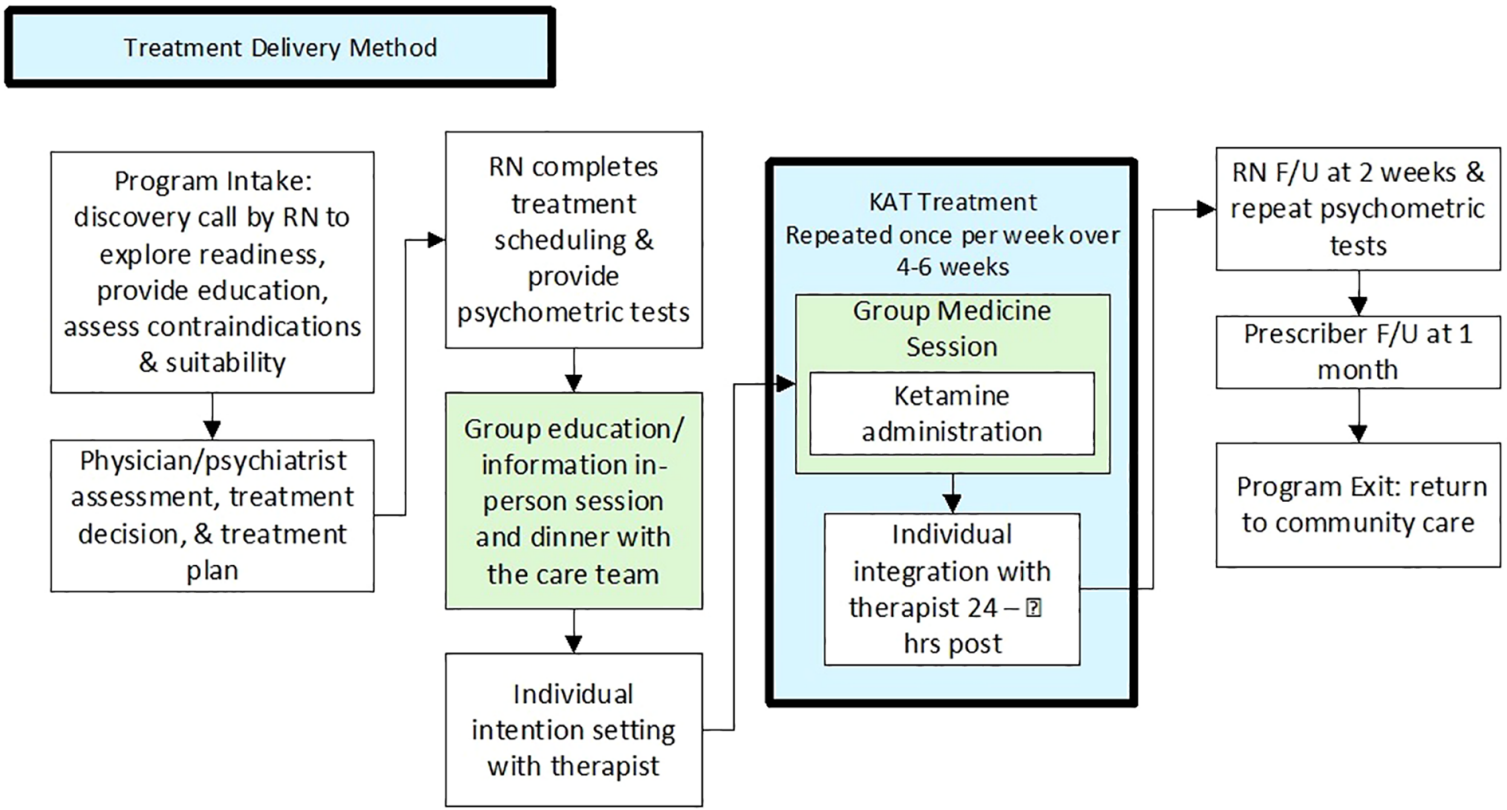
Treatment Delivery Method.

The clinical design and delivery of ketamine-assisted therapy (KAT) was collaboratively developed by the ATMA CENA interdisciplinary treatment team in partnership with Siksika Health Services (SHS) (see **Figure 1**). The regulated health professionals involved included a psychiatrist, nurse practitioner (NP), registered nurses (RNs), an advanced care paramedic, and a registered psychologist. Clinical decision-making, including participant screening, dosing, medical oversight, and psychological support, in alignment with policy, regulation and treatment standards identified by Alberta Health and the College of Physicians and Surgeons of Alberta. The process was designed and conducted transparently with SHS leadership awareness to ensure cultural alignment and participant safety.

Participant recruitment and intake: Participant recruitment occurred through a self-referral process following education sessions provided to SHS Indigenous and non-Indigenous staff. The intake pathway began with an RN interaction to assess readiness, identify contraindications, and provide preliminary education. Each participant then completed a virtual medical and mental-health assessment with the NP and/or psychiatrist, who evaluated program suitability, confirmed the absence of contraindications, and established initial and booster dosing ranges. A formal treatment plan and prescription for ketamine was issued by the psychiatrist following intake, the RN contacted participants to schedule intention-setting sessions, group medicine days, integration sessions, and to coordinate transportation.

Orientation and pre-treatment measures: Participants received standardized pre-treatment psychometric packages via email, which were repeated after program completion (see **Table 1**). One week before treatment initiation, participants attended an in-person group orientation event that included sharing a meal, educational content, informed consent review and completion, introduction to the nursing team and psychologist, and all participated in a check-in sharing circle allowing for story telling and asking questions aimed at building comfort, psychological safety, trust, and group cohesion amongst staff and participants.

**Table 1:** Data from psychometric tests.

	GAD-7		PHQ-9		PCL-5	
Client	Pre	Post	Pre	Post	Pre	Post
A	7-mild	9-mild	11-moderate	5-mild	30	12
B	7-mild	1-non-indicated	10-moderate	0-non-indicated	20	7
C	5-mild	7-mild	5-mild	5-mild	29	3
D	4-non-indicated	1-non-indicated	4-non-indicated	4-non-indicated	10	4
E	19-mod-severe	3-non-indicated	15-severe	3-non-indicated	48	7
F	*No data did not do screens was diagnosed both PTSD and GAD by intake NP - did not do post measures either					
G	*No data did not do screens was diagnosed mild PTSD and GAD by intake NP – did not post measures – dropped out after 1tx					

*Note: of clients ABCDE – one had 3 KAT, the rest had 4-6KAT

PCL-5 scoring description.

- 19-29: little to no severity of symptoms of PTSD.

- 28-29: some symptoms of PTSD.

- 30-44: moderate to moderately high PTSD symptoms.

- 45-85: high severity of symptoms.

Treatment: The week after orientation KAT commenced with a single individual intention setting session with the therapist, followed by a series of six group medicine sessions offered to participants, each medicine session was followed by an individual integration session. Post program, participants were offered up to three additional integration sessions to support their well-being and smooth transition back to their original care team if needed. Ketamine was administered intramuscularly (IM), chosen for its consistency, bioavailability, and dose accuracy relative to sublingual routes. Dosing generally ranged from 0.5–2.0 mg/kg, with no observed differential effects between Indigenous and non-Indigenous participants. Initial dosages and dose adjustments were made collaboratively by the psychiatrist, psychologist, and ketamine-administering nurses following clinical rounds after each medicine day. Determinants of initial dose and dose changes included prior anesthetic response, concurrent medications, physical health considerations, and participant-reported effects (e.g., nausea). Two participants, one Indigenous and one non-Indigenous, experienced nausea on at least one medicine day, attributed to insufficient food intake around treatment sessions.

Staffing model and safety: Each medicine day was staffed by three RNs or by two RNs and an advanced care paramedic, providing an approximate 2:1 participant-to-provider ratio. A structured post-session reflection circle facilitated voluntary sharing; an RN remained present to provide grounding or clinical support as needed. All participants were required to have a designated support person for pickup after each medicine session.

Psychological support structure: A registered psychologist with experience in rural First Nation communities and in psychedelic-assisted therapy oversaw all intention-setting and integration sessions. The psychologist attended the first two medicine days onsite to support participant comfort and address early fears or uncertainties. Although the intended model was for integration sessions to occur in person within 48 hours of treatment, several participants required virtual or telephone-based sessions occurring within four days of treatment when in-person attendance and timeliness was not possible. Four of the six participants completed at least two of the post program integration sessions.

Treatment frequency, schedule, and cultural considerations: The treatment schedule was co-designed by ATMA CENA and SHS to reflect both conventional KAT protocols and participant needs. Week one contained two medicine days, mirroring common Alberta KAT practices; subsequent weeks followed a once-weekly schedule in response to participant feedback of needing more time for personal integration between medicine sessions and disruption to daily responsibilities. After the fourth medicine session, a three-week break was introduced based on SHS and participant feedback indicating desire to attend the upcoming Siksika Nation’s annual cultural events, including ceremonies, a fair, music festival, and Pow Wow. Several participants attended cultural activities during this period. Though atypical for KAT protocols, incorporating this pause aligned with SHS leadership’s cultural wisdom and participant autonomy. This integration of cultural engagement is hypothesized to have contributed to increased trust, comfort, and mutual respect and understanding between participants, the non-Indigenous treatment team, and SHS leadership team.

Final treatment schedule:

- Week 1: Treatment#1 Tuesday; Treatment#2 Thursday.
- Week 2: Treatment#3 Tuesday.
- Week 3: Treatment#4 Tuesday.
- Three-week break for cultural events and integration.
- Week 4: Treatment#5 Tuesday.
- Week 5: Treatment#6 Monday.

Post-treatment care: Following completion of the program, participants received a follow-up call from the RN and were re-issued psychometric measures via email. Prior to discharge, each participant also completed a virtual follow-up with an NP to determine ongoing health needs and ensure continuity of care. Participants then exited the program and returned to their usual community supports.

Culturally responsive design and community involvement: the pilot was co-developed using a culturally grounded, trauma-informed framework shaped by input from the First Nation community leadership and the clinical team. Program components incorporated culturally meaningful elements akin to healing circles, guided storytelling, collective intention-setting, and opportunities for ceremonial practices where appropriate and comfortable for participants. These elements informed both the therapeutic container and group process structure.

Sharing circles, not formal traditional healing circles, were used to foster relational safety, reciprocity, and shared understanding, core components of Indigenous wellness traditions. Storytelling was integrated into intention-setting and post treatment group sharing or report-out sessions as a culturally congruent method for meaning-making and emotional processing. The treatment environment prioritized autonomy, respect, and psychological safety, with flexibility to accommodate cultural practices identified by participants or community partners.

Although the medicine administration followed clinical best practices for KAT, the surrounding preparation and integration were deliberately adapted to reflect Indigenous knowledge systems and community preferences, recognizing their essential role in promoting trust, reducing cultural barriers, and supporting healing.

## Data analysis and results

Demographics: Given the small cohort (N = 7) drawn from a single First Nation community, reporting detailed demographic characteristics such as age, gender or diagnoses introduces a meaningful risk of participant re-identification. As such, demographic reporting was limited to group-level identity only (Indigenous vs. non-Indigenous). Overall, participants were adults who varied in age and gender, and presented with mental health concerns typical for referral to KAT, including anxiety, depressive symptoms, and trauma-related distress. The small sample size supports caution but allows meaningful interpretation of outcomes, findings and insights to support future care provision in this population.

Treatment completion and follow-up: all 7 participants were present for orientation, intention setting session with psychologist, and for treatment day #1. One participant did not return after treatment #1 due to experiencing diabetes related side effects. This participant did complete one integration session but did not complete further follow-up or post evaluation. The remaining group treatments had one or two participants miss at least one, which resulted in group sizes ranging from 4–6 participants in each group treatment. One participant completed all six treatments offered and attended all integration sessions including two follow-up sessions. Of the six who remained in treatment, the range of total completed ketamine therapy sessions was 3-6. All participants completed integration sessions after each medicine session. The participant that completed three ketamine treatments did not do their final integration session but did do a follow-up support session with the psychologist on the phone due to unforeseen life circumstances preventing further participation in treatment or follow-up.

Participant attendance and psychometric completion rates were as follows:

- 1 Indigenous participant attended 1 medicine session; no post-treatment measure or evaluation completed.
- 1 Indigenous participant attended 3 medicine sessions; no post-treatment measures or evaluation completed.
- 1 Indigenous participant attended 6 medicine sessions; completed all post-treatment and evaluation measures.
- 1 Indigenous participant attended 5 medicine sessions; completed all post-treatment and evaluation measures.
- 1 non-Indigenous participant attended 4 medicine sessions; completed all post-treatment and evaluation measures.
- 2 non-Indigenous participants attended 5 medicine sessions; completed all post-treatment and evaluation measures.

Quantitative Findings: **Table 1** details our quantitative findings. Psychometric tests used by the psychiatrist for suitability and pre-testing and conducted by the nurse practitioner for post-testing included: the Generalized Anxiety Disorder-7 (GAD-7) (29), Patient Health Questionnaire-9 (PHQ-9) (30), and the PTSD Checklist – civilian version (PCL-5) (31). Initially the tests were sent out electronically with intent that they would be completed independently prior to the appointments with the psychiatrist and later the NP. However, due to low completion rates 5 of the 7 pre-tests were completed over the phone with the support of an RN and the completed post tests were done over the phone with the NP. Five of the seven participants completed post-treatment psychometric assessments. 4 of these participants completed the post-psychometric testing in one month, with the fifth participant completing the post testing after two months. Across these five individuals, reductions were observed in symptoms of anxiety (GAD-7), depression (PHQ-9), and PTSD-related distress (PCL-5).

Most participants demonstrated shifts from moderate symptom categories at baseline toward mild or minimal symptom ranges post-treatment. Participants with elevated PCL-5 scores at intake showed reductions that moved them into lower severity bands, indicating decreased PTSD-related symptoms. Although the magnitude of improvement varied, the overall pattern showed consistent symptom reduction across measures. Upon review of the quantitative results between participants who completed four treatment sessions versus those who completed five or six, no observable differences in outcomes were found between participants. This suggests that participants experienced measurable benefits even when treatment engagement varied.

Qualitative findings: **Table 2** details the qualitative findings. The self-evaluation data were completed and submitted anonymously by five participants. Responses indicated that the program was experienced as safe, supportive, and therapeutically beneficial. The following counts represent how many participants mentioned each theme explicitly in their evaluation:

**Table 2:** Survey data.

Question	Ptcp* A	Ptcp B	Ptcp C	Ptcp D	Ptcp E
The KAT helped my mental health	4	3	4	4	4
I am glad I participated in the program	4	3	4	4	4
I would have preferred not to have done KAT	1	1	1	1	1
I feel I am worse of mentally after doing KAT	1	1	1	1	1
I feel I got what I needed out of my KAT	3	3	2.5	3	4
Overall experience with KAT impacted me negatively	1	1	2	1	1
Overall experience with KAT impacted me positively	4	3	4	4	4
I believe I benefitted from KAT	4	4	4	4	4
I have less irritability	3	3	3.5	3	4
My level of energy went up	3	2	3.5	3	3
My sleep improved	4	2	3.5	3	3
I am more aware of my body and felt a sense of emotion in my body since KAT	4	2	3.5	4	3
My mood improved after treatments	3	3	3.5	4	4
My anxiety improved after treatments	4	3	3.5	4	4
I feel I have benefitted spiritually from KAT	3	3	3.5	4	4
I feel I have benefitted emotionally from KAT	3	3	3.5	4	4
I would like to have more KAT	4	2	4	4	4
I made meaning of KAT experience in integration	3	3	4	3	4
Able to take what learned in integration and apply it in my life to create some changes	3	3	3.5	3	4
Preferred to do only ketamine and not integration	3	1	1	1	1
Integration was an important part of KAT	3	4	4	4	4
I found integration sessions helpful	3	4	4	4	4
I benefitted from doing integration after ketamine	4	4	4	4	4
Prefer integration therapist present after KAT at debrief	2	4	2.5	4	4
Prefer integration therapist present when under KAT	2	2	2.5	2.5	3
Prefer integration therapist present at beginning KAT	2	3	2.5	2.5	4
I had enough time in integration sessions	4	3	4	4	2
I was able to be open with my integration therapist about my KAT experience	3	4	4	4	4
I was able to trust my integration therapist	3	4	4	4	4
I felt I could trust my tx team to handle anything I would experience	4	2	4	4	4
I felt comfortable with tx team prior to beginning KAT	4	2	4	4	4
I felt understood and seen by my nurse during screening and intake	4	3	4	4	4
I was informed about risks of KAT prior to tx	4	2	4	4	4
I had opportunity to ask questions and how it will impact me prior to participating in program	4	3	4	4	4
I was informed of precautions to take prior to tx	4	3	4	4	4
I was informed/educated about health benefit of KAT	4	4	4	4	4
It was important to meet tx nurses ahead of time prior to KAT	4	4	4	2	4
The group orientation was important to prepare me for tx	3	3	4	4	4
I felt the medical team was prepared on tx days	4	2	4	3	3
I would prefer to do KAT individually	4	3	4	2	3
I would prefer to do ketamine in a group	2	2	4	3	3
Group debrief after medicine was important	2	3	4	4	4
Preferred to have been in tx room by myself	3	3	4	2	2
Felt comfortable being in group tx room	3	3	1	4	3

*Responses by 5 of 7 participants to post=program survey. Ptcp = participant.

Scale: 1. Strongly disagree, 2. Somewhat disagree, 3. Somewhat agree, 4. Strongly agree.

- trust in and feeling safe with the treatment team (5/5);
- benefit and usefulness of medicine days - insight, emotional processing, or symptom relief (5/5);
- benefit and usefulness of integration sessions - clarifying meaning and applying insight) (5/5); and
- improvements across several wellbeing domains:
  - energy, mood stability, reduced irritability (4-5)
  - better sleep and decreased anxiety (3-4/5), and
  - enhanced spiritual grounding, connection and emotional wellbeing (3/5).

## Discussion: practical implications and lessons learned for future application

This pilot demonstrated that a collaboratively designed ketamine-assisted therapy (KAT) model, developed with and led by Siksika Health Services (SHS), is both feasible and clinically impactful for Indigenous and non-Indigenous participants. The “heal the healers” design, which recruited SHS staff as the initial cohort, enabled rapid engagement, clear communication pathways, and meaningful community leadership in shaping the treatment model. Seven individuals enrolled (four Indigenous and three non-Indigenous); six continued beyond the first session, receiving three to six treatment days across the ultimately five-week program, with flexibility incorporated to accommodate personal, cultural, and community needs.

Attrition in one Indigenous participant after the first session underscored an important clinical and operational consideration: the high prevalence of diabetes among Indigenous populations and the necessity of treatment-specific nutritional guidance. This participant experienced significant nausea after not eating the evening before or morning of treatment, despite guidance that light intake was permitted. This highlights a clear implication for prescribing physicians and nurses: KAT protocols for Indigenous clients must explicitly integrate diabetes management, fasting timelines, and culturally appropriate pre-treatment nutritional instructions to mitigate avoidable adverse effects and reduce attrition.

**Figure 2:**
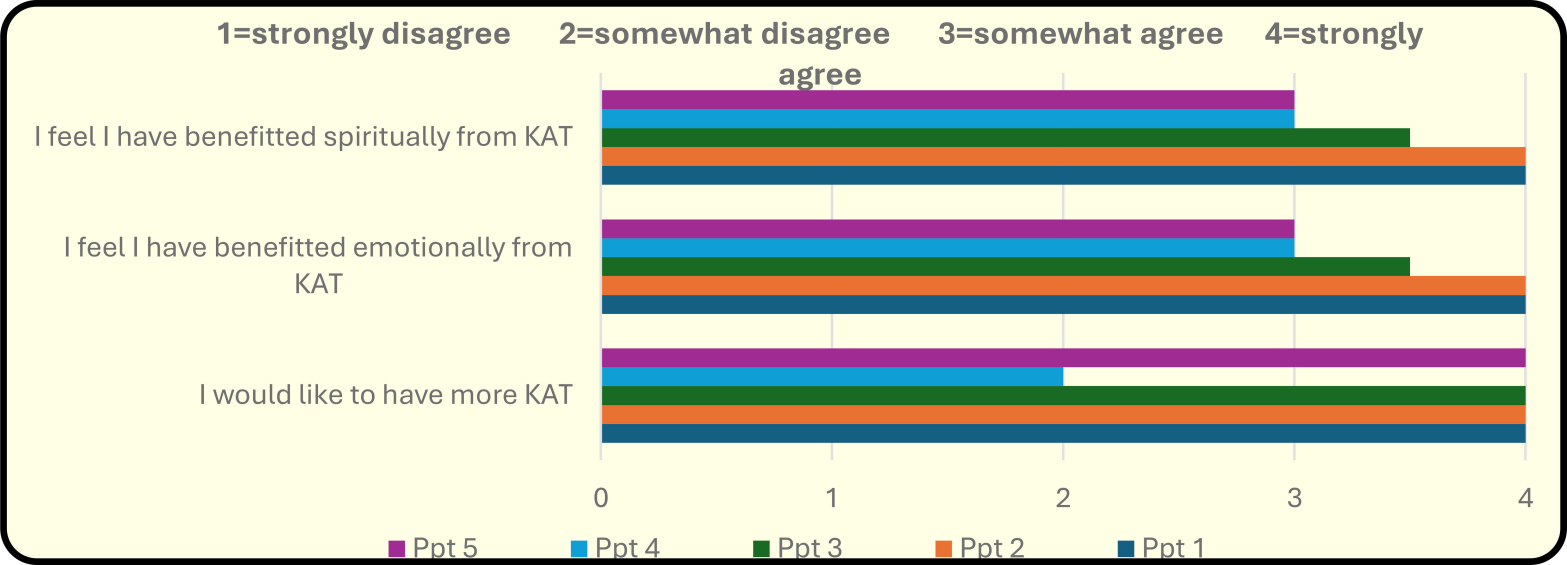
Theme Benefit of KAT.

Across the remaining six participants, outcomes were consistently positive, reflected in psychometric improvements (**Table 1**), self-report data (**Table 2**, **Figure 2**), and thematic patterns emerging during integration sessions. One Indigenous participant who completed three treatments was unable to continue due to urgent family matters and could not complete post-treatment psychometrics or the client evaluation; however, they did complete two integration sessions following their final medicine day, allowing for qualitative capture of their experience. Overall, all six participants demonstrated comparable benefit, suggesting that partial completion of the dosing series did not preclude meaningful therapeutic gain. Reasons for missed sessions differed by group: Indigenous participants more commonly cited social determinants affecting themselves or family members, while non-Indigenous participants cited competing work or leisure commitments. These distinctions reinforce the importance of operational flexibility, the capacity for make-up sessions, and awareness that socioeconomic and structural realities may shape engagement differently across participant groups.

A central contributor to safety, trust, and comfort was the culturally responsive design developed jointly by SHS and the ATMA CENA clinical team. Participants—both Indigenous and non-Indigenous—reported feeling understood and supported by the treatment team. This finding is particularly notable in light of longstanding health inequities experienced by Indigenous peoples within clinical environments, including disparities in access, quality of care, and relational safety. The First Nation–led decision to include Indigenous and non-Indigenous participants in the same treatment group, to determine set and setting, and to guide the pacing and rhythm of the program represents a structural challenge to entrenched systemic inequities in healthcare delivery.

**Figure 3:**
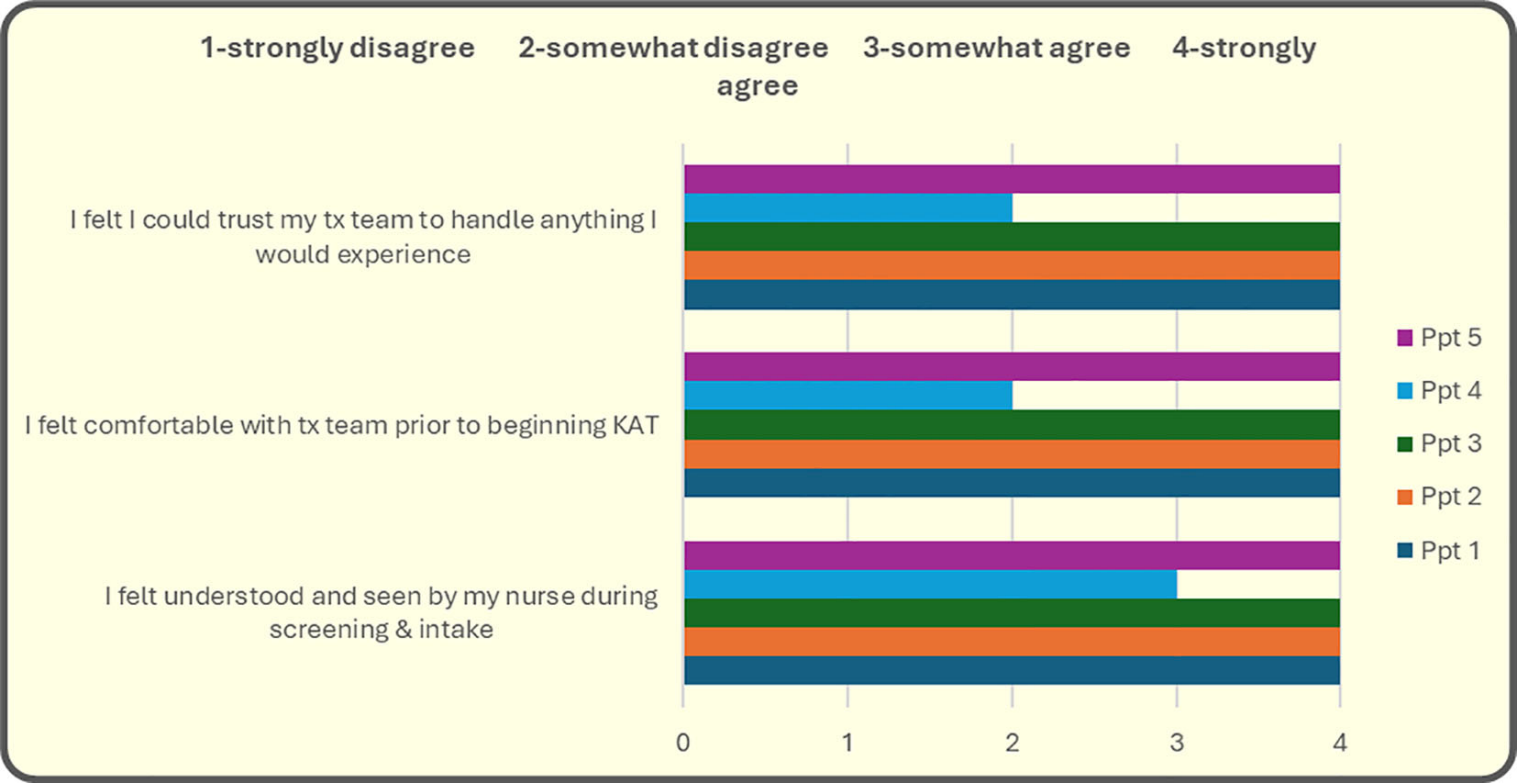
Theme Comfort With Team.

Culturally grounded design choices appeared to have direct therapeutic relevance. SHS established the set and setting, selected group composition, guided preparatory education, and created an environment in which cultural practices and community values shaped the treatment experience (see **Figure 3**). The introduction of a three-week pause—coinciding with traditional community gatherings, ceremonies, and Pow Wow events—allowed participants to engage voluntarily in cultural activities mid-treatment. Although a break of this duration is atypical in conventional KAT protocols, participant feedback suggested that this period of cultural reconnection deepened trust, supported emotional regulation, and enhanced meaning-making during subsequent integration sessions. This finding underscores the importance of Indigenous autonomy in program design and suggests that embedding cultural rhythms directly into clinical timelines may enhance therapeutic impact rather than diminish it.

**Figure 4:**
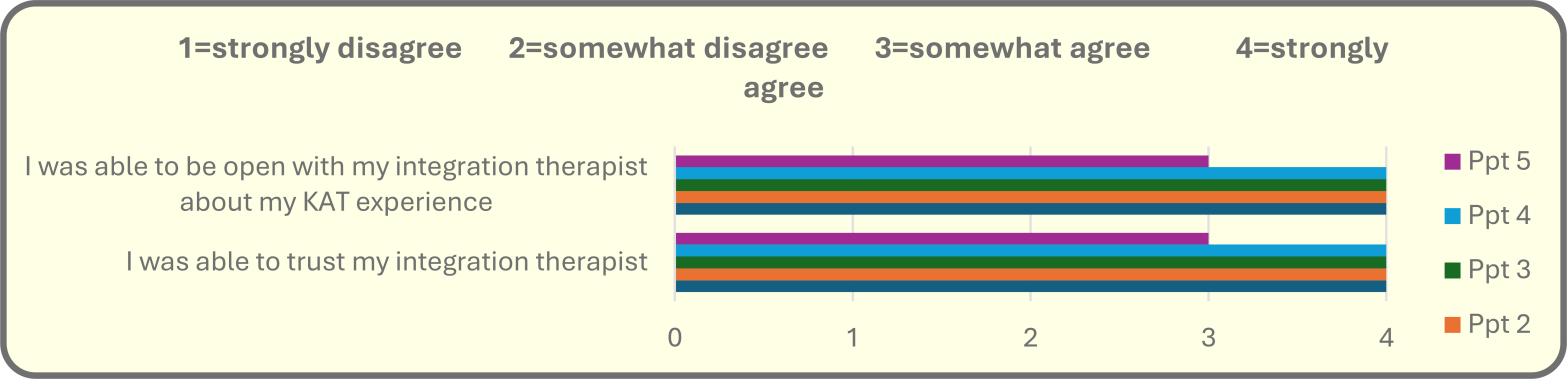
Theme Comfort With Therapist.

The therapist’s role emerged as a critical mechanism for safety, continuity, and accurate interpretation of participant experiences (see **Figure 4**). All participants completed intention-setting with the same psychologist, who had experience working in First Nation communities and formal training in psychedelic-assisted therapy. This continuity extended across medicine and integration sessions and supported participants in processing experiences that included grief, loss, fears related to work and family expectations, spiritual connection, ancestral presence, and reconnection with land and culture. For Indigenous clients, certain visions or symbolic experiences—such as ancestral guidance, animal protectors, or spiritual encounters shaped by Christian teachings linked to residential school histories—require careful interpretation grounded in cultural humility. Avoiding the imposition of Western psychological frameworks onto culturally specific meaning-making processes was essential. The therapist’s cultural competence and continuity were likely central contributors to the trust and positive outcomes reported.

From an operational perspective, several challenges were identified. Technology-based communication strategies were largely ineffective; participants were more responsive to direct phone calls or text messages, necessitating staffing flexibility. Maintaining consistent clinical staffing across all medicine and integration sessions was difficult, raising concerns about potential fragility in therapeutic rapport—particularly for Indigenous participants with historical mistrust of health systems. Early introduction of all providers helped mitigate this risk. Integration sessions did not always occur within the ideal neuroplastic window of 24–72 hours post-medicine due to scheduling conflicts and life demands; nonetheless, participants continued to report subjective benefit. Several participants required rescheduled or virtual integration sessions and additional post-program support, indicating that standard post-treatment integration models may be insufficient in community-based or culturally diverse settings.

The observed reductions in symptoms of depression, anxiety, and PTSD are consistent with emerging evidence supporting the efficacy of ketamine-assisted therapy across diverse clinical populations, including those with treatment-resistant conditions. Prior studies have demonstrated rapid and sustained symptom improvement following KAT, even with variable dosing schedules (20, 21). Within this pilot, comparable benefits were observed across participants completing four to six treatment sessions, suggesting that flexibility in treatment delivery may be compatible with meaningful clinical outcomes. Importantly, the strong qualitative themes of trust, safety, emotional regulation, and spiritual connection align with broader Indigenous mental health literature emphasizing the centrality of relational, cultural, and spiritual dimensions of healing.

Participants consistently reported improvements in emotional regulation, self-awareness, connection to culture or spirituality, and overall well-being. These qualitative findings, together with psychometric improvements, suggest that culturally adapted KAT holds promise as a meaningful mental health intervention within Indigenous contexts. At the same time, the pilot illuminated persistent system-level barriers, including stigma surrounding psychedelic therapies, limited leadership familiarity, funding constraints, and challenges related to scalability. Expanding participant numbers, extending follow-up periods, and embedding KAT within longer-term community wellness strategies will be critical for future evaluation.

More broadly, this pilot contributes to growing evidence that psychedelic-assisted therapies can be implemented in culturally aligned, community-driven ways that enhance trust, engagement, and outcomes for Indigenous clients. When Indigenous leadership guides program structure, prepares the therapeutic environment, and shapes delivery, KAT can function not only as a symptom-reduction intervention but as a holistic healing process supporting emotional, cultural, and spiritual well-being. The lessons from this pilot provide actionable insights for designing larger-scale programs, informing health policy, and establishing equitable pathways for collaboration across public, private, and federal systems.

Ultimately, effective implementation of KAT in Indigenous communities requires integration of cultural knowledge, clinician expertise, flexible operational design, and sustained follow-up. By centering Indigenous leadership and respecting community-determined approaches to healing, future programs can build on this foundation to improve mental health outcomes and reduce longstanding disparities in care.

## Acknowledgment of any conceptual or methodological constraints

Real world program development and implementation typically sit in the realm of quality improvement. While quality improvement holds more narrowly defined goals than implementation science and less stringent methodology, both are acknowledged as sharing similarities and a common focus to improve clinical practice (32). As such, both approaches were considered when indicating pilot project constraints.

The pilot program encountered several methodological and conceptual limitations that should be considered when interpreting the findings. One of the primary constraints was the small sample size, which limited the power and generalizability of the results. While the data collected provides valuable preliminary insights, future implementation projects should aim for a larger and more diverse participant group to enhance the reliability of the findings.

Another challenge was the reliance on self-reported measures for assessing treatment outcomes. While qualitative and quantitative assessments provided useful information, self-reported data is inherently subject to bias, including social desirability and recall bias. To strengthen future evaluations, incorporating objective biomarkers or third-party clinical assessments would provide a more comprehensive understanding of the impact of Ketamine-Assisted Therapy (KAT) on mental health outcomes.

Additionally, logistical and scheduling challenges affected the consistency of integration sessions and the completion of psychometric testing and surveys. Some participants were unable to attend follow-up sessions within the optimal neuroplasticity window, potentially influencing the long-term effectiveness of the therapy. Additionally, the timing, approach and support for quantitative and qualitative data capture can be evolved based on what was discovered in the pilot. Future implementations should consider structured follow-up protocols that ensure accessibility and adherence to recommended post-treatment integration practices and data capture.

Cultural considerations also played a significant role in shaping the pilot. While efforts were made to align the KAT program with Indigenous traditions, there remains a need for deeper exploration of how Indigenous worldviews and healing practices can be further integrated into psychedelic-assisted therapies. Continuous engagement with Indigenous Elders, knowledge keepers, and community members is essential for ensuring that these therapies remain respectful and relevant to cultural contexts.

Finally, funding and resource constraints posed limitations on the scope and scalability of the pilot program. Securing sustainable funding and policy support will be crucial for expanding KAT within Indigenous healthcare systems. Further research, particularly longitudinal studies, will be required to understand the lasting effects of KAT and establish best practices for its integration into mental health services.

Despite these constraints, this pilot program provides valuable initial evidence supporting the feasibility and benefits of KAT within an Indigenous healthcare framework. Addressing these limitations in future implementations and program development will help enhance the efficacy, accessibility, and cultural appropriateness of psychedelic-assisted therapies for Indigenous communities.

## Data Availability

The raw data supporting the conclusions of this article will be made available by the authors, without undue reservation.
